# Surgical Treatment of Far Lateral Lumbar Disc Herniation: Outcomes of the Safe and Simple Midline Approach

**DOI:** 10.7759/cureus.27907

**Published:** 2022-08-11

**Authors:** Mustafa Kaya, Emrah Keskin, Davut Ceylan, Tibet Kacira, Özlem Kitiki Kacira

**Affiliations:** 1 Department of Neurosurgery, Sakarya University Education and Research Hospital, Sakarya, TUR; 2 Department of Neurological Surgery, Zonguldak Bülent Ecevit University, Zonguldak, TUR; 3 Department of Radiology, Sakarya University Education and Research Hospital, Sakarya, TUR

**Keywords:** microdiscectomy, surgery, midline, disc hernia, far lateral

## Abstract

Objective

Surgery for far lateral lumbar disc herniation (FLLDH) hernias is different than surgery for median and paramedian disc hernias. Our study offers a minimally invasive surgical technic for far lateral disc herniations.

Methods

The results of the midline surgical approach in 18 patients diagnosed with FLLDH were evaluated retrospectively.

Results

A total of 18 patients (7 females, 11 males), with a mean age of 57.9±9.4 years (range: 35-71 years), were included in the analyses. Three patients (16.7%) had lesions at the left L3-L4 level, six patients (33.3%) on the left L4-L5 level, five patients (27.8%) on the right L3-L4 level, and four patients (22.2%) on the right L4-L5 level. All patients had low back and leg pain. These complaints completely regressed after surgery.

Conclusion

This study presents a review of a consecutive series of patients who underwent minimally invasive surgery for FLLDH using a midline approach.

## Introduction

Far lateral lumbar disc herniation (FLLDH) is defined as a disc herniation located on the sagittal plane laterally to the medial wall of the pedicle [1-3]. This definition is also made as extra-canalicular, extreme lateral, and post-foraminal disc hernia and describes the same pathology [2]. Especially after the use of technologies such as magnetic resonance imaging (MRI) and high-resolution computed tomography (HRCT), FLLDH could be defined more and approximately 7%-12% of lumbar disc hernias were reported as far lateral disc herniations [3-5]. Approximately, 75% of far lateral discs are seen at the L3-4 and L4-5 disc levels. It is most common at the L4-5 level and then at the L3-4, L5-S1, and L2-3 levels, respectively [3,5-6].

Typically, FLLDH compresses the nerve root at the same level, rather than at the level below [7]. In brief, the exiting nerve root is compressed and displaced [8-9]. Most of the patients with FLLDH present to the hospital with unilateral severe radicular pain. Often, the femoral stretch test and the straight leg stretching test are positive. Decrease in muscle strength and tone, atrophy, loss of reflex, and hypoesthesia due to the compression of the affected root and ganglion are other symptoms and signs that can be seen. It was reported that neuropathic pain developed more frequently in patients whose symptoms were prolonged [10-11].

For median disc herniations, the classic median approach was first described by Mixter and Barr in 1934; however, this traditional technique is insufficient for FLLDH [12-13]. For FLLDH, several conventional techniques have been described in the literature as extending medial exposure to medial facetectomy, total facetectomy, and the trans-pars technique and extraforaminal exposure [11-14]. However, hemilaminectomy is performed as the lateral expansion of the classical median approach, and the partial or total laminectomies added to it cause radicular pain due to segmental instability and asymmetric disc collapse [15-17]. More recently, the preferred method is increasingly shifting toward the minimally invasive surgery (MIS) and modified paramedian approaches, which intend to protect the lamina and the facet has been commonly used [1,4].

Our study aims to describe the extraforaminal microdiscectomy for FLLDH by midline incision, using the Taylor retractor and routine lumbar disc herniation (LDH) tools. As with other surgical approaches for FLLDH, root damage, residual disc, cerebrospinal fluid leakage, and neuropathic leg pain due to manipulation of the dorsal root ganglion are possible complications [7,15-16]. In the present study, the results of 18 patients were retrospectively evaluated, and the obtained experiences and the mid-term surgical outcomes of FLLDH were shared.

## Materials and methods

This study is a retrospective study performed on 18 patients who underwent FLLDH operation within 3 years (2017 to 2020) at Sakarya University Faculty of Medicine. The study started with the approval of the Sakarya University Faculty of Medicine Ethics Committee (02.03.2021 date, number 2021/153).

**Figure 1:**
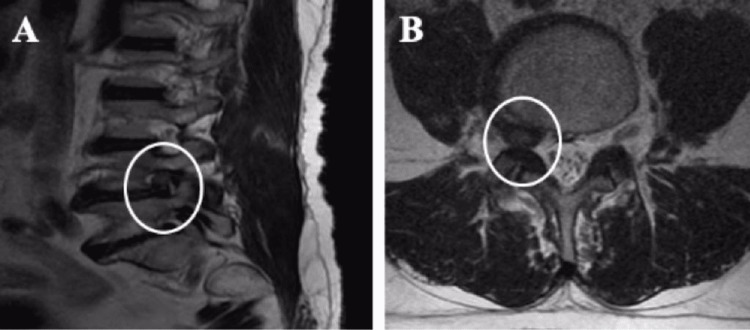
Sagittal and axial T2 image of right L4-L5 FLLDH on preoperative lumbar MRI FLLDH: far lateral lumbar disc herniation

All patients were admitted to the hospital with radicular pain. In addition to pain, 16 patients had numbness and tingling, and all patients had varying degrees of motor deficit. The clinical information of the patients and their pre/postoperative MRI and/or CT images were obtained from the hospital archive. Adult patients with FLLDH causing root compression on lumbar MRI (Figure 1), severe leg pain (with low back and/or low back pain) lasting at least one month, Lasegué positivity on neurological examination, and motor and/or sensory deficits were included in the study. Patients who had previous spinal surgery for any other reason or for lumbar disc herniation (LDH) were excluded from the study. In addition, patients with spinal instability, spinal infection, and spinal malignancy in their preoperative evaluations were not included in the study. Before surgery, patients received conservative treatment for at least three weeks. Early visual analog scale (VAS) and Oswestry Disability Index (ODI) tests (preop and postop first day) were obtained from the archives of the neurosurgery department while late VAS, ODI tests (sixth month), and flexion-extension lateral lumbar X-ray radiographs were obtained from the archives of the outpatient clinic.

Surgical procedure

**Figure 2:**
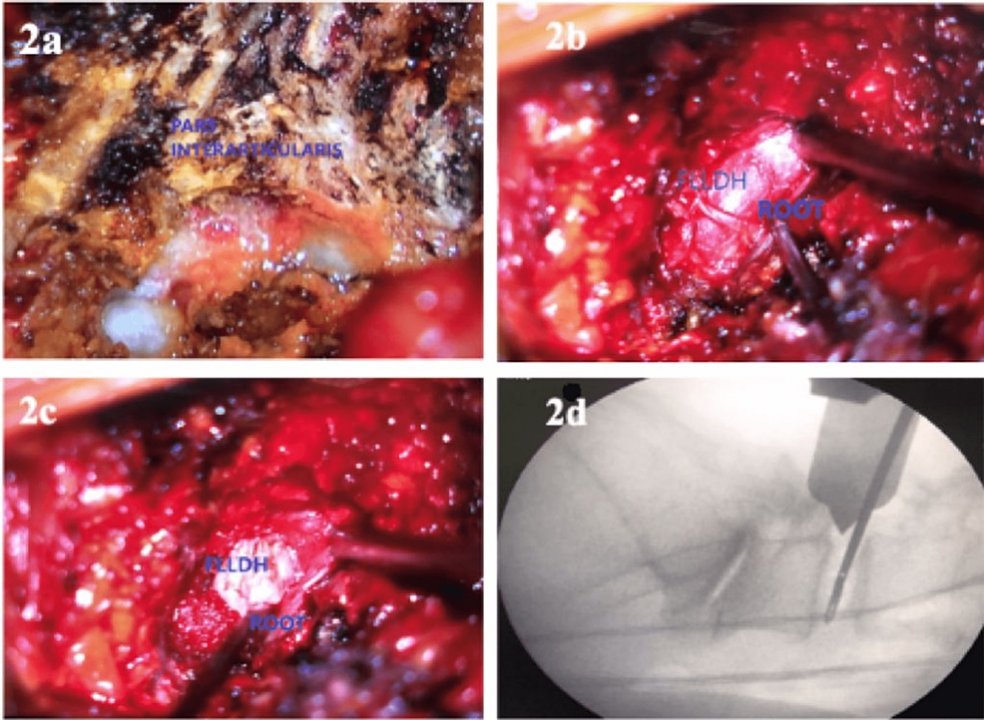
Operational images pars interarticularis (2a) and a far lateral disc herniation in L4-L5 (2b, 2c) with the exiting nerve root under compression (2b, 2c). The C-arm fluoroscopy image of the microdiscectomy performed by reaching the disc distance from the supralateral (2d)

The distance is determined precisely by C-arm fluoroscopy by inserting a needle without making a skin incision. An approximately 3 cm midline skin incision is made. The fascia opens again at the midline and the paravertebral muscles are stripped subperiosteally. Before placing the retractor, a fluoroscopy image is taken again and the classical Taylor retractor distance is confirmed and placed on the lateral side of the facet joint. After this stage, the superior edge of the transverse process, the lateral edge of the pars interarticularis, and the facet joint complex are seen under the operating microscope (Figures 2a-2c).

**Figure 3:**
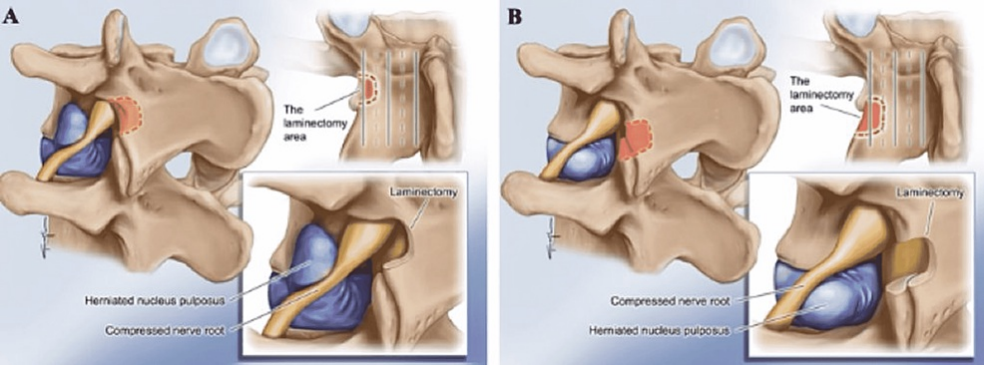
Midline approach for far lateral disc herniations A: Diagram illustrating a far lateral disc herniation cranial migration fragment (purple) with exiting nerve root impingement. The area of the pars interarticularis recommended for drilling is indicated by the red dashed line; B: Diagram illustrating a far lateral disc herniation fragment (purple) located at the disc distance with exiting nerve root impingement. The area of the pars interarticularis recommended for drilling a larger laminectomy area is indicated by the red dashed line.

The bone is drilled according to the location of the disc fragment, which is extruded. If the disc herniation is toward the cranial (often migrated towards the cranial), the lateral surface of the pars interarticularis is drilled (Figure 3A). However, if the disc herniation is at the disc distance, the pars lateral is less resurrected and the facet joint lateral surface (especially the superior facet lateral) is resurrected more (Figure 3B). Then, the intertransverse ligament is found at the upper border (margin) of the transverse process and released with the help of a Kerrison Rongeur. Exiting root and dorsal root ganglion are seen more clearly after the adipose tissue surrounding them is cleaned. After the root is removed from the superior and lateral, the extruded disc piece is reached and discectomy is performed after this stage. The patient is mobilized after six hours and discharged within 24 hours postoperatively. Microdiscectomy can be performed when necessary, by reaching the disc distance from the supralateral (Figure 2d).

Statistical analysis

The descriptive statistics of continuous variables were presented using mean and standard deviation and minimum and maximum values, and those of categorical variables were presented with frequency and percent. The comparisons of continuous data between dependent groups were conducted with Friedman non-parametric analyses of variances, and the post hoc analyses in cases of an overall significance were tested with the Wilcoxon signed-ranks test. The statistical significance level in overall comparisons was a p-value lower than 0.05 (Type-I error level <5%, which was modified according to the number of pairwise comparisons in post-hoc analyses (Bonferroni correction). All analyses were conducted using SPSS 25 (IBM Corp., Armonk, NY) software.

## Results

A total of 18 patients (7 females, 11 males), with a mean age of 57.9±9.4 years (range: 35-71 years), were included in the analyses. Six patients (33.3%) had lesions at the left L4-L5 level, five patients (27.8%) on the right L3-L4 level, four patients (22.2%) on the right L4-L5 level, and three patients (16.7%) on the left L3-L4 level. The mean preoperative symptom duration was 3±1.2 weeks, and the mean operation time was 56.8±10.4 minutes. The mean amount of intraoperative bleeding was recorded as 52.8±13.9 cc. Patients were followed up for a mean duration of 13.6±5.5 weeks (Table 1). In our study, complications such as root injury, residual disc, cerebrospinal fluid leakage, or hematoma were not observed in any of the cases. No signs of instability were observed in the follow-up imaging of the patients.

**Table 1:** General demographic and clinical characteristics of patients VAS: visual analog score; ODI: Oswestry Disability Index

	Mean±SD/n (%)	Min-Max
Age (Years)	57.9±9.4	35-71
Sex		
Female	7 (38.9)	
Male	11 (61.1)	
Level of hernia		
Left L3-L4	3 (16.7)	
Left L4-L5	6 (33.3)	
Right L3-L4	5 (27.8)	
Right L4-L5	4 (22.2)	
Preoperative symptom time (week)	3±1.2	1-5
Operation time (min)	56.8±10.4	43-75
Amount of bleeding (cc)	52.8±13.9	32-85
Postoperative follow-up (months)	13.6±5.5	7-26
VAS		
Preoperative	7.9±1.1	6-9
Postoperative	2.7±0.8	2-4
Postoperative 6^th^ month	0.6±0.8	0-2
ODI		
Preoperative	54.6±6.5	46-66
Postoperative	19.6±3	16-26
Postoperative 6^th^ month	9.8±2.9	6-16

**Figure 4:**
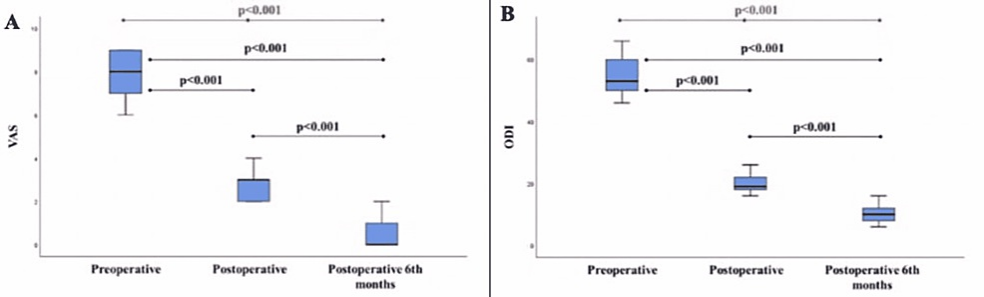
The VAS (A) and the ODI (B) assessments in the preoperative and postoperative periods VAS: visual analog score; ODI: Oswestry Disability Index

The VAS levels decreased from preoperative 7.9±11 to postoperative 2.7±0.8 in the postoperative period, and to 0.6±0.8 in the postoperative sixth month. The overall change was statistically significant (p<0.001, Table 1), and the post hoc analyses revealed a gradual decrease through follow-ups (Figure 4A).

The preoperative median value of the ODI was 54.6±6.5, which decreased to 19.5±3 during the postoperative period, and to 9.8±2.9 in the postoperative sixth-month assessments (p<0.001, Table 1). The pairwise post hoc comparisons revealed a gradual decrease through follow-ups (Figure 4B).

## Discussion

Various techniques have been defined in FLLDH surgery since 2000. Microscopic surgery through a working channel, percutaneous endoscopic discectomy (PED), microscopic intertransverse discectomy, extraforaminal approach, and the trans-pars techniques are more popular [11,18-19]. The present study offers a review of a consecutive series of patients who underwent MIS surgery for FLLDH, receiving the minimal lateral pars and the minimal facet lateral face with a midline approach. It is believed that the benefits of the MIS approach described in this study and by other authors emphasize the minimal soft tissue dissection and bone removal applicable for FLLDH at any level associated with minimal postoperative pain and no more than an overnight hospital stay [18-19]. All spinal surgeons are familiar with this method and there is no need for extra hardware or surgical instruments.

The herniated or sequestered disc materials are usually displaced cephalad, with only 20% at the level of the affected intervertebral space; 80% of patients present with cranial migration [8-9,15,20-21]. A total of 15 (84%) patients in the present study had upper migration and three (16%) had FLLDH in the disc distance. Drilling the lateral side of the pars interarticularis is sufficient to remove the disc in patients with migration from the disc distance to the cranial. However, if the herniation is at disc distance, the disc herniation can be easily reached by drilling the pars interarticularis slightly or by drilling the lateral facet surface without drilling.

**Figure 5:**
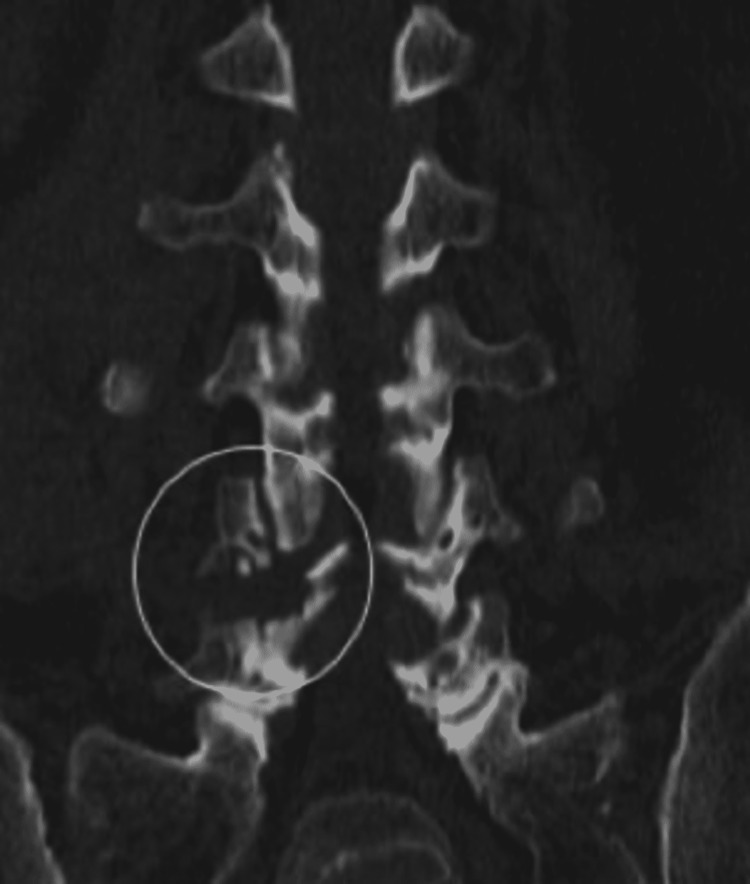
Postoperative lumbar coronal CT shows partial resection of the pars inter articulation (white circle)

In a biomechanical study on sheep spine, Sarı et al. compared the vertebrae in which they resected 50% and resected 25% lateral to the pars interarticularis [16]. The 50% resected group was found to be more susceptible to lateral bending [16]. With partial pars resection, the amount of bone resection does not exceed the lateral 1/4th width of the pars as demonstrated in one of our patient’s postoperative CT scans (Figure 5). No instability or spondylolisthesis was observed in the follow-up of the patients whose facet lateral and pars lateral were removed. None of the patients suffered instability or spondylolisthesis warranting a second procedure.

Compared to FLLDHs with median and paramedian LDH, they are seen in older patients and the mean age range of incidence is 55-56 [1,17]. The average age in the current study was 57.9 years (Table 1). Especially in older patients, the length of the surgical time and the amount of bleeding during surgery are important. The mean duration of surgery in FLLDH cases was reported to be 41-70 minutes [1,3]. However, in the present study, the surgical time was 56.8 minutes (Table 1). The amount of bleeding during surgery was reported to be 124-131.32 ml for FLLDH [3]. In the current study, there was a bleeding of 52.8 ml (Table 1).

Among MIS techniques, PED may be defined as the most minimally invasive technique [17]. But Aydın et al. compared the PED and microscopic extraforaminal discectomy (MEFD) techniques applied in the same group [17]. They reported severe complications in PED, particularly root injury. They suggested that this multiplicity of complications was due to the variations of Kambin's triangle [22]. They reported that they preferred MEFD because of its lower complication rate. In the surgery performed with a midline incision in the context of root damage, the root and ganglion are encountered early when the pars and/or facet joint laterals are turned, and this may be an advantage to reduce the rupture.

Chronic compression of the dorsal root ganglion by FFLDH is a significant cause of neuropathic pain. If surgical treatment is delayed for FLLDH, the risk of persistent neuropathic pain may increase [10]. In the present study, medical treatment was tried to be kept short in the preoperative period (mean 3±1.2 per week, Table 1). Moderate neuropathic pain persisted in two of 18 patients until the fourth month postoperatively. Gabapentin treatment in these patients was terminated at the end of the fourth month.

Our study is limited by the small number of cases and the short follow-up period. Despite these limitations, the existence of different criteria for evaluating our results allowed us to obtain reliable results.

## Conclusions

Surgery for FLLD hernias is different than surgery for median and paramedian disc hernias. Enclosing the pars and facet lateral with the midline approach is a minimally invasive method that can be applied without the need for extra equipment and surgical hand tools.
